# Cage-templated synthesis of highly stable palladium nanoparticles and their catalytic activities in Suzuki–Miyaura coupling†
†Electronic supplementary information (ESI) available: Detailed experimental materials, general synthetic procedures, TEM images, and spectral characterization data. See DOI: 10.1039/c7sc03148c


**DOI:** 10.1039/c7sc03148c

**Published:** 2017-11-09

**Authors:** Li Qiu, Ryan McCaffrey, Yinghua Jin, Yu Gong, Yiming Hu, Hongliang Sun, Wounjhang Park, Wei Zhang

**Affiliations:** a School of Materials Science and Engineering , Yunnan Key Laboratory for Micro/Nano Materials & Technology , Yunnan University , 650091 Kunming , China; b Department of Chemistry and Biochemistry , University of Colorado at Boulder , CO 80309 , USA . Email: Wei.Zhang@colorado.edu; c Department of Electrical , Computer and Energy Engineering , University of Colorado at Boulder , CO 80309 , USA

## Abstract

We report the controlled synthesis of small palladium nanoparticles (PdNPs) with narrow particle size distribution (1.8 ± 0.2 nm) using an organic molecular cage as a template. The well-defined cage structure and thioether anchoring groups inside the cavity are critical for the formation of narrowly distributed PdNPs, offering a confined organic molecular environment and guiding PdNP nucleation and growth. The resulting encapsulated PdNPs are resistant to agglomeration and stable in solution exposed to air at room temperature. When provided with a protective cage shell with minimum surface coverage, such PdNPs are capable of catalyzing organic reactions, showing high catalytic activity in Suzuki–Miyaura coupling reactions.

## Abstract 2

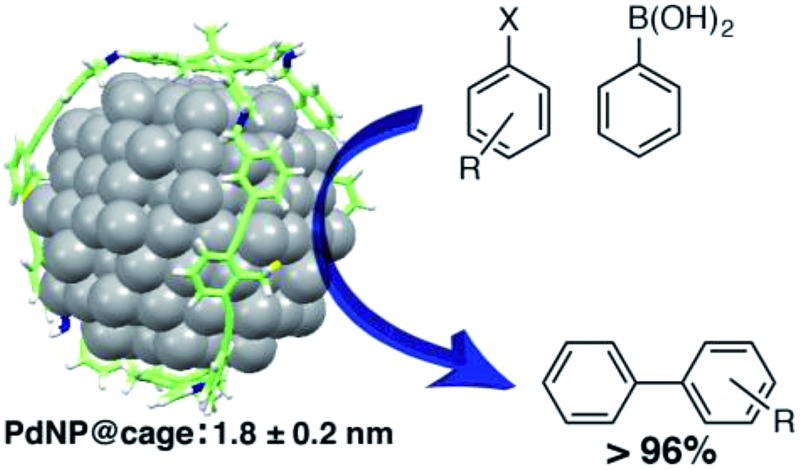
Cage-templated synthesis of narrowly distributed palladium nanoparticles (1.8 ± 0.2 nm) and their high catalytic activity in Suzuki–Miyaura coupling reactions are reported.

## Introduction

Metal nanoparticles (NPs) have been widely applied in various disciplines of modern sciences, including catalysis,^1^–^6^ diagnostic imaging,^7^–^10^ sensing,^7^,^11^ magnetic recording,^12^ electronics^13^ and optics.^14^ These materials often exhibit particular physical and chemical characteristics arising from their small size and high surface-to-volume ratio, which are distinct from those of bulk materials.^15^ The properties of metal NPs highly depend on their size, shape and composition,^16^,^17^ thus the synthesis of narrowly distributed particles of a specific structure and composition has become an important research area in nanoscience. Various solution phase methods have been developed for the synthesis of nanoparticles, many of which are based on surface capping ligands and dendritic architectures.^3^,^18^–^20^ Recently, the synthesis of monodisperse nanoparticles has advanced through the use of closed-shell, hollow “ship-in-bottle” structures, such as protein cages,^21^ supramolecular DNA assemblies,^22^,^23^ and metal-coordination complexes,^24^ which confine the particles to a specific size and shape. However, the size-controllable preparation of small and narrowly dispersed nanoparticles remains challenging.

Our group has been interested in exploring shape-persistent 3-D organic molecular cages as templates to control encapsulation and growth of nanoparticles. The recent advent of dynamic covalent chemistry (DCvC)^25^–^28^ has enabled facile large-scale synthesis of shape-persistent 3-D organic molecular cages, which have attracted tremendous attention as viable candidates for applications such as carbon capture and fullerene separation.^29^–^34^ With well-defined and permanently rigid pore structures, such a cage template^35^–^37^ can offer a protecting shell with minimum surface coverage, which would be advantageous compared to conventional small organic ligands or macromolecular ligands that form thick, insulating layers on the nanoparticle surface.^38^,^39^ Herein, we report robust organic cage-templated synthesis of stable, highly soluble, and narrowly distributed palladium nanoparticles (PdNPs). Their catalytic application in Suzuki–Miyaura cross-coupling reactions was also explored.

## Results and discussion

**Scheme 1:**
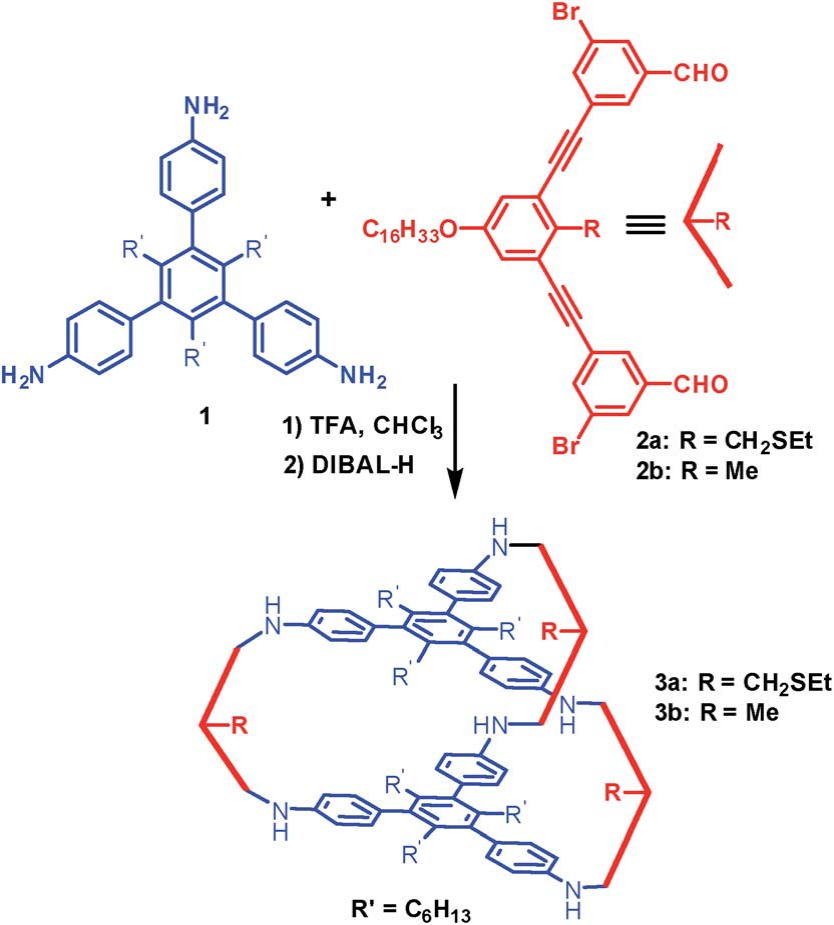
The synthesis of cages **3a** and **3b**.

Cage **3a** with a large internal void and pendant interior thioether anchoring groups was synthesized through dynamic imine chemistry from triamine **1** and dialdehyde **2a** in one step (Scheme 1).^35^ The analogous cage **3b** with methyl groups instead of thioether groups was synthesized for comparison. Both cages were characterized by ^1^H and ^13^C NMR, GPC, and MALDI-MS. Palladium nanoparticles were then prepared *via* a two-phase liquid–liquid approach adapted from Brust *et al.* in the presence of cage molecules.^40^–^42^ A solution of a phase-transfer reagent, tetraoctylammonium bromide (TOAB), in CH_2_Cl_2_ was added to an aqueous solution of K_2_PdCl_4_ (5 equiv.) and stirred until the aqueous layer was colorless, indicating that all of the PdCl_4_^2–^ was transferred to the organic phase. A solution of cage **3a** (1 equiv.) in CH_2_Cl_2_ was added to the above biphasic mixture and stirred for 45 minutes. The orange-red color deepened and the mixture was subsequently reduced with an aqueous solution of sodium borohydride (190 equiv., rt) resulting in a dark brown organic phase with no precipitation, which indicates the efficient reduction of Pd^2+^ and further stabilization of PdNPs by cage molecule **3a**. After extraction using CH_2_Cl_2_, the resulting PdNP@**3a** solution was dried over sodium sulfate, precipitated out from ethanol, and characterized by UV-vis, ^1^H NMR (DOSY), HR-TEM and energy-dispersive X-ray spectroscopy (EDS).

**Fig. 1:**
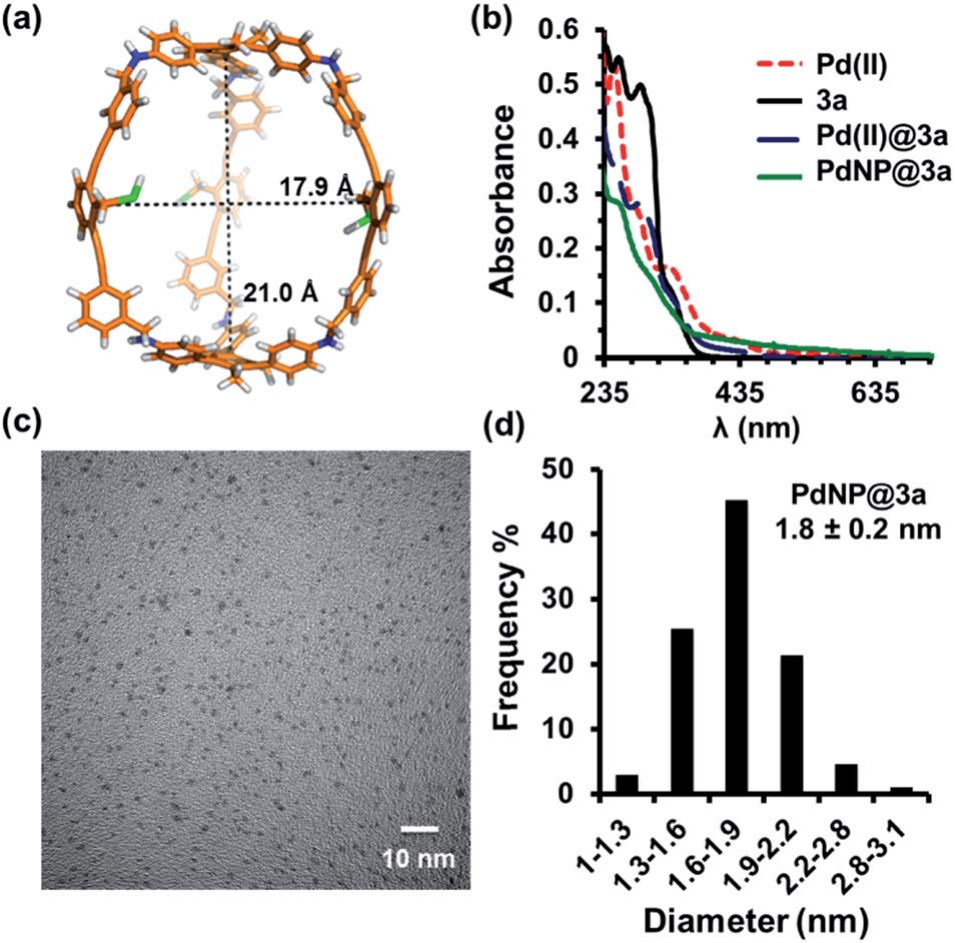
(a) Calculated cavity size of fully extended cage **3a**; (b) UV-vis absorption spectra of cage **3a** and the palladium complexes in CH_2_Cl_2_; (c) HRTEM micrographs (scale bar 10 nm) of PdNP@**3a**; (d) Size distribution of the PdNP@**3a** complex.

The UV-vis absorption spectra of the solution before and after reduction are shown in Fig. 1b. In the absence of cage **3a**, the absorption spectrum of tetrabutylammonium tetrachloropalladate(ii) in CH_2_Cl_2_ shows absorption peaks at *λ* = 250 nm and 320 nm arising from ligand-to-metal charge-transfer transitions (Pd^II^, red line, Fig. 1b). After the addition of cage **3a** to the Pd^II^ solution, the charge-transfer bands decreased in intensity and the absorption of **3a** appeared as a shoulder band around 275 nm (Pd^II^@**3a**, blue line, Fig. 1b). Complete reduction of PdCl_4_^2–^ to Pd^0^ and the formation of PdNPs was supported by the absence of bands from 300–500 nm (PdNP@**3a**, green line, Fig. 1b), which correlates well with results previously reported in the literature.^43^,^44^

The diameter and size distribution of the resulting PdNP@**3a** were then analyzed by TEM micrographs (Fig. 1c, S3†). A solution of PdNP@**3a** in CH_2_Cl_2_ was drop cast onto carbon-coated 300 mesh copper grids (CF300-Cu) and allowed to air dry before the measurements. The TEM image (Fig. 1c) shows well-dispersed PdNPs with an average size of 1.8 nm (over 500 particles counted), which matches well with our computational models showing an internal cavity size of 1.8–2.1 nm (Fig. 1a and d). The formation of such small PdNPs agrees well with the absence of a plasmon peak in the UV-vis spectrum. Energy-dispersive X-ray spectroscopy (EDS) (Fig. S5†) also confirmed unambiguously the presence of metallic palladium. MALDI-TOF and ESI-MS spectra of PdNP@**3a** show the presence of various multiply-charged Pd_5_@**3a**, Pd_12_@**3a**, Pd_18_@**3a**, Pd_22_@**3a**, and Pd_38_@**3a**, supporting the complexation of PdNPs with the cage. These PdNP@**3a** complexes are stable and highly soluble in common organic solvents.

Consistent with our previous findings on AuNP@**3a**,^35^ substantial broadening and shifting of not only the protons of the thioether group but also all of the aromatic protons of the cage skeleton were observed in the ^1^H NMR spectra (Fig. S2†) of the above PdNP@**3a** complex (with a small amount of phase-transfer agent TOAB to maintain good solubility in benzene). This indicates that the cage shell is likely to be tightly wrapped around the PdNP and experiences restricted mobility. ^1^H diffusion-ordered spectroscopy (DOSY) NMR performed on both the free cage **3a** and PdNP@**3a** under the same temperature and concentration (Fig. S6 and S7†) gave very similar diffusion coefficients of (2.5 ± 0.5) × 10^–10^ m^2^ s^–1^ and (2.4 ± 0.5) × 10^–10^ m^2^ s^–1^ respectively, which indicates the similar size and shape of the free cage and PdNP@**3a**. It therefore also supports the notion that PdNPs rest inside the cage cavity instead of aggregating on the cage surface.

It is expected that thioether groups inside the cage cavity provide a preferential nucleation site for Pd, and further deposition in the spatially confined cavity would provide PdNPs of a similar size to the cage. Additionally, encapsulation of PdNPs in isolated cavities would also prevent their agglomeration and improve the chemical and thermal stability. Control experiments in the absence of cage **3a** led to the formation of a black precipitate (Fig. S4†), supporting the notion that the presence of cage **3a**, which contains six amino groups and three thioether groups, is necessary for the growth, size control, and stabilization of PdNPs. In order to understand exactly what structural requirements are necessary for PdNP growth, we performed additional control experiments in the presence of cage **3b**, where the only structural difference is that the three thioethers are replaced with methyl groups. We again observed the complete precipitation of PdNPs. A further control experiment, in which only the thioether side piece **2a** was used as the stabilizing ligand, also led to similar aggregation and precipitation, further suggesting that the closed cage and multidentate interaction involving thioether groups is necessary for PdNP-cage stabilization.

With the successful formation of stable PdNP@**3a**, we next explored the use of PdNP@**3a** in homogenous catalysis. The advances in novel catalytic technologies for more efficient chemical transformations have arguably been some of the most significant developments in the history of modern science. As compared to their bulk counterparts, nanoparticle-based catalysts often exhibit unique and enhanced catalytic properties due to their high surface area, size reduction, and shape variation.^45^–^54^ Since palladium-mediated catalysis has been widely applied and shown great synthetic power in current organic chemistry,^55^ as a proof-of-principle, we chose to use one of the most popular carbon–carbon (C–C) bond formation reactions, the Suzuki–Miyaura cross-coupling reaction, to evaluate the catalytic activity of PdNP@**3a**. The coupling reactions were carried out using phenylboronic acid (1.5 equiv.) and PdNP@**3a** (0.01 equiv.) along with various aryl iodides and aryl bromides bearing a variety of functional groups. All reactions were performed in a mixture of toluene/H_2_O (10 : 1, v/v) using Na_2_CO_3_ (3 equiv.) as a base. The catalytic activity of PdNP@**3a** was first investigated using phenylboronic acid and aryl iodides at 100 °C under microwave heating (Table 1, entries 1 and 2). The reaction progress was monitored *via* TLC and the crude reaction mixtures were analyzed by ^1^H NMR spectroscopy. Although conversion was good after 10–15 minutes of reaction, we found that increasing the reaction time to 30 minutes gave almost quantitative yields. To broaden the scope of the PdNP@**3a** catalyst, the same conditions were applied to a series of aryl bromide substrates using a temperature of 140 °C. We obtained the desired coupling products in almost quantitative yields in most entries. The analysis of the crude reaction mixture by ^1^H NMR shows little evidence of impurity formation during the coupling except in entry 5, which was seen to contain 1–2% biphenyl by-product.

**Table 1:** Suzuki–Miyaura coupling of various aryl halides using PdNP@**3a** and Pd(PPh_3_)_4_^*a*^ ^,^^*b*^

Entry	Aryl halide	Product	Yield [%]	
			PdNP@**3a**	Pd(PPh_3_)_4_
1	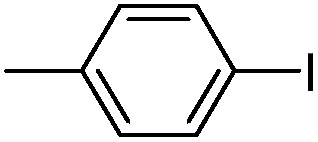	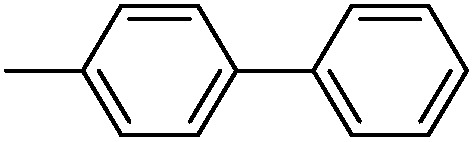	99	86, (99^*c*^, 98^*d*^, 22^*e*^)
2	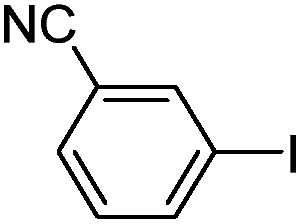	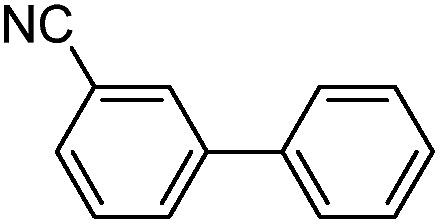	>99	81
3	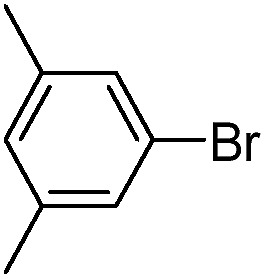	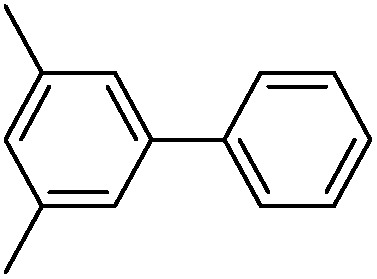	96	85
4	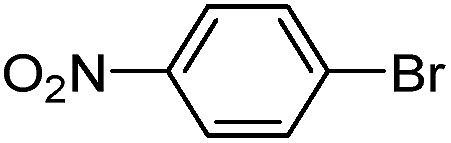	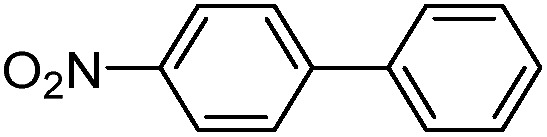	>99, >99^*f*^	78, 40^*f*^
5	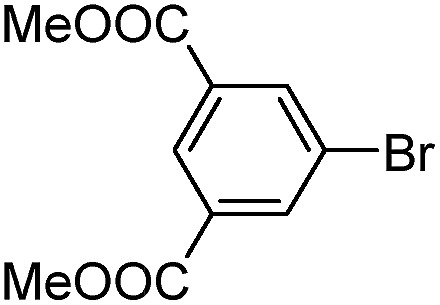	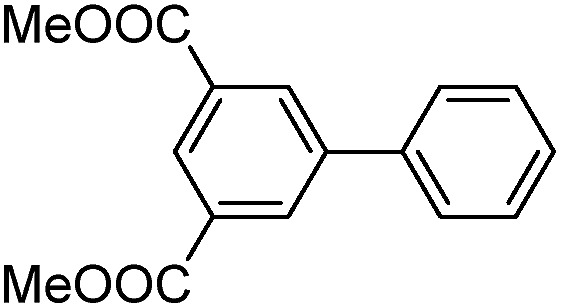	>99	73
6^*g*^	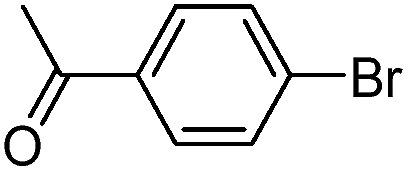	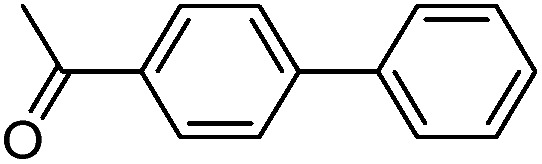	99 ± 0.4	75, (78^*c*^, 72^*d*^, 20 ± 2.4^*e*^)

^*a*^Reaction conditions: aryl halide (0.057 mmol), phenylboronic acid (0.087 mmol), Na_2_CO_3_ (0.17 mmol), Pd catalyst (0.57 μmol, 1.0 mol%).

^*b*^Yields are based on ^1^H NMR analysis of the crude products.

^*c*^For Pd(PPh_3_)_2_Cl_2_ catalyst.

^*d*^For Pd_2_(dba)_3_.

^*e*^For Pd/C (5%).

^*f*^After exposure of the catalyst to air for 2.5 h.

^*g*^5 repeats with a standard deviation for both PdNP@**3a** and Pd/C (5%).

As a comparison, the commercially available palladium (0) tetrakis catalyst Pd(PPh_3_)_4_ was used under the same conditions. We found the conversion to the desired products could only be achieved in yields of 73–85% (Table 1) after 30 min. Although we observed only a modest increase in overall yields using PdNP@**3a**, we found that a major advantage of this cage-based catalyst is its ability to be stored in solution under atmosphere over a period of several hours with no decrease in activity, thus highlighting its superb stability under ambient conditions. Such stability is remarkably superior to palladium tetrakis catalyst, which must be stored under inert atmosphere with very limited exposure to air. When both catalysts are stored in solution for 2.5 hours (open to air), the activity of PdNP@**3a** remained the same while the activity of Pd(PPh_3_)_4_ was decreased to 40% (Table 1, entry 4), further corroborating the stability of the nanocatalyst. In addition to Pd(PPh_3_)_4_, three other commercially available palladium catalysts, Pd(PPh_3_)_2_Cl_2_, Pd_2_(dba)_3_ and Pd/C were employed for comparison. As shown in entries 1 and 6, while both Pd(PPh_3_)_2_Cl_2_ and Pd_2_(dba)_3_ gave comparably high yields to PdNP@**3a** for iodotoluene, PdNP@**3a** showed higher activity for brominated substrates. When compared to the commercial nanoparticle Pd/C (5%), PdNP@**3a** exhibited a much better performance, not only with higher yields (99% *vs.* ∼20%, entries 1 and 6) but also with a higher reaction rate: the reaction between 4-iodotoluene and phenylboronic acid catalyzed by PdNP@**3a** produced 4-methyl-1,1′-biphenyl with an 80% conversion after 6 min of reaction with a turnover frequency (TOF) of 800 h^–1^, which is about 6 times greater than that of Pd/C catalysts (TOF: 130 h^–1^) under the same conditions. Meanwhile PdNP@**3a** showed very good reproducibility for the reaction between phenylboronic acid and 4′-bromoacetophenone, with a standard deviation (in reaction yield) of only 0.4% in five repeats (entry 6). The catalytic efficiency of PdNP@**3a** can be attributed to both its high solubility and largely unpassivated surface, which presumably allows for easy substrate diffusion to the nanoparticle surface.^37^ Although precipitation of palladium black was observed occasionally after removal of the reaction vessel from the microwave reactor (which is probably due to the high temperature breaking the bonding interaction between PdNP and the cage), interestingly, the recycled palladium catalyst could be reused at least 3 more times in the coupling reaction without loss of yield (Table S1†), which supports the high reactivity of PdNP@**3a**.

As previously discussed, traditional synthetic methods for PdNPs rely heavily on classic organic supports (*e.g.* polymers,^56^ dendrimers,^57^–^59^ micelles,^60^*etc.*) to prevent NP agglomeration at the colloidal stage. It has been reported that some dendrimer-stabilized PdNPs show good stability and excellent catalytic activity. For example, Astruc and co-workers demonstrated that PdNPs stabilized by dendritic nanoreactors have high catalytic activity (TOF = 4.5 × 10^4^ h^–1^) toward the Suzuki–Miyaura reaction of 1,4-bromonitrobenzene and phenylboronic acid.^59^ However, in general, there is a compromise between the stability of the resulting NPs and the available surface accessibility for substrate activation and transformation.^61^,^62^ For instance, the El-Sayed group found that although G4 dendrimer-encapsulated PdNPs were more stable than polymer-stabilized PdNPs, the catalytic activity was in fact lower.^63^ In our cage-based supports, not only can the particle size be predetermined by the cage cavity size, but further stability and resistance to agglomeration can also be achieved *via* the well-defined closed architecture of the cage. This is in great contrast to dendritic architectures where increases in generation lead to steric crowding on the dendrimer periphery and subsequent lowering of the activity of the PdNP.^64^ Our study successfully demonstrates the use of organic cage molecules as suitable colloidal chemistry platforms for the controllable synthesis and stabilization of nanometer-sized catalytically active metallic nanoparticles. Due to their highly accessible, well-defined surface morphology and long shelf-life in solution, cage-encapsulated nanoparticles show great promise as convenient and active catalysts.

## Conclusions

In summary, we report the synthesis and stabilization of ∼1.8 nm-sized PdNPs within the cavity of an organic cage molecule, which show excellent catalytic activity in the Suzuki–Miyaura reaction. The incorporation of metallic nanoparticles within the cavities of well-defined, discrete molecular cage molecules as novel catalytic supports is attractive given their stability and size programmability within the interior cage dimensions. Our cage-template approach will pave new avenues in the development of significantly improved or even novel catalysts for various chemical transformations through the use of different metallic nanoparticle centers. In addition, we envision that such an approach could provide a powerful platform for controlled growth of novel nanostructured materials, which can be used in a range of nanotechnologies, including nanocatalytic applications.

## Conflicts of interest

There are no conflicts to declare.

## Supplementary Material

Supplementary information
