# Supplementary material for: Cage-templated synthesis of highly stable palladium nanoparticles and their catalytic activities in Suzuki–Miyaura coupling
Source: Chem Sci. 2017 Nov 9;9(3):676–80. doi: 10.1039/c7sc03148c (PMC5869600; doi:10.1039/c7sc03148c)
Supplement: Supplementary file 1 [file SC-009-C7SC03148C-s001.pdf]

Electronic Supplementary Material (ESI) for Chemical Science.
This journal is © The Royal Society of Chemistry 2017

Cage-Templated Synthesis of Highly Stable Palladium Nanoparticles and Their Catalytic Activities in Suzuki-Miyaura Coupling

Li Qiu,[ab‡] Ryan McCaffrey,[b‡] Yinghua Jin,[b] Yu Gong,[b] Yiming Hu,[b] Hongliang Sun,[a] Won Park[c] and Wei Zhang[*b]

[a]School of Materials Science and Engineering, Yunnan Key Laboratory for Micro/Nano Materials & Technology, Yunnan University,650091 Kunming, China

[b]Department of Chemistry and Biochemistry, University of Colorado at Boulder, CO 80309, USA. E-mail: Wei.Zhang@colorado.edu

[c]Department of Electrical, Computer and Energy Engineering, University of Colorado at Boulder, CO 80309 USA.

[‡]These authors contributed equally.

Supplementary Information

Table of contents

1. Materials and General Methods

2. Experimental Procedures

3. GPC Graph of Cages **3a** and **3b**

4. Transmission Electron Microscopy (TEM)

5. Energy-dispersive X-ray spectroscopy (EDS)

6. Diffusion NMR experiment

7. NMR spectra of compounds

8. References

1. Materials and general methods

All commercially available reagents and solvents were used as received, unless noted otherwise. CH_2Cl_2 and toluene were purified by MBRAUN solvent purification system. All reactions were carried out under dry nitrogen in flame dried glassware, unless noted otherwise. After workup, all solvents were removed by rotary evaporator. Unless other indicated, the purity of the compounds was ≥ 95 % based on ^{1}H NMR spectral integration. Flash column chromatography was performed using 100-150 times weight excess of 32-63 μm silica gel from Dynamic Absorbants Inc. Fractions were analyzed by TLC using TLC silica gel F254 250 μm precoated-plates from Dynamic Absorbants Inc. Gel permeation chromatorgraphy (GPC) was performed using a Viscotek GPCmaxTM, a Viscotek Model 3580 Differential Refractive Index (RI) Detector, a Viscotek Model 3210 UV/VIS Detector and a set of two Viscotek Viscogel columns (7.8 x 30cm, 1- MBLMW-3078, and 1-MBMMW-3078). GPC calibration was done using monodisperse polystyrene standards and THF was used as the eluent at 30 ºC. UV-vis absorption measurements were recorded with an Agilent 8453 spectrophotometer. Matrix Assisted Laser Desorption Ionisation (MALDI) mass spectra were performed on the Voyager-DE™ STR Biospectrometry Workstation in linear mode using a sinapic acid matrix. High-resolution mass spectra were performed on a Waters SYNAPT G2 high definition mass spectrometry system. Energy-dispersive X-ray spectroscopy (EDS) was performed using a JEOL JSM-6480 scanning electron microscope with an elemental detection lower limit of carbon. All microwave reactions were conducted under dry nitrogen in flame dried glass tube using Discover SP microwave from CEM. ^{1}H and ^{13}C NMR spectra were obtained from either an Inova 500 or Bruker 300 spectrometer. $CHCl_3$ (7.27 ppm) was used as an internal

reference in ^{1}H NMR, and $CHCl_3$ (77.23 ppm) for ^{13}C NMR. NMR data is reported in the following order: chemical shift, multiplicity (s, singlet; d, doublet; t, triplet; q, quartet; m, multiplet), coupling constants (J, Hz), number of protons.

2. Experimental procedures

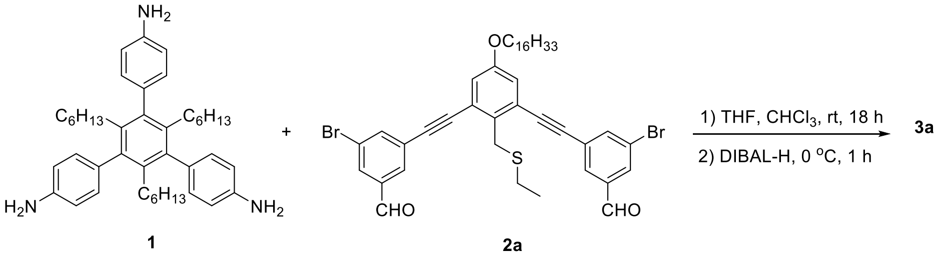

Cage 3a: For the synthesis of Cage **3a**, please refer to our previous publication.[1] The physical data for cage **3a**: ^{1}H NMR (C_6D_6, 500 MHz) δ 7.60 (s, 6H), δ 7.46 (s, 6H), 7.36-7.17 (m, 24H), δ 6.41 (d, J = 8.5 Hz, 12H), δ 4.40 (t, J = 6.0 Hz, 6H), δ 3.70 (d, J = 5.3 Hz, 12H), δ 3.29 (m, 12H), δ 2.52 (s, 12H), δ 2.14 (q, J = 7.4 Hz, 6H), δ 1.93 (m, 6H), δ 1.65 (m, 8H), δ 1.51 (m, 16H), δ 1.31 (m, 74H), δ 1.11-0.86 (m, 80H), δ 0.79 (t, J = 7.1Hz, 18H); ^{13}C NMR (101 MHz, C_6D_6): δ 161.03, 145.75, 142.72, 140.00, 139.48, 134.32, 134.08, 132.62, 131.10, 130.08, 128.67, 125.33, 122.69, 117.61, 113.10, 111.54, 92.50, 87.15, 74.63, 46.89, 34.56, 32.04, 32.04, 31.05, 30.77, 29.93, 29.85, 29.66, 29.54, 26.57, 24.96, 22.83, 22.31, 14.12, 14.07; MS (MALDI) Cal'd. for $C_{213}H_{264}Br_6N_6O_3S_3$ $[M]^+$ 3531.50; Found 3530.78.

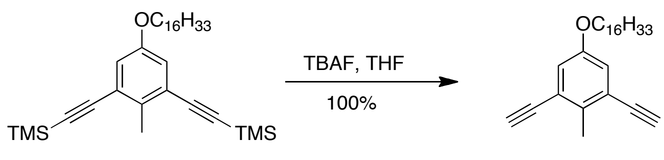

4 **5**

Compound 5: To a Schlenk tube were added compound **4** (500 mg, 0.95 mmol) and THF (15 mL) under nitrogen atmosphere. The solution was cooled to 0 °C using an ice bath. A solution of TBAF (1 M in THF, 1.9 mL, 1.9 mmol) was added dropwise at 0 °C. The darkened solution was warmed to rt and stirred for 30 min. The solvent was removed by rotary evaporation, and the crude product was passed through a short column of silica gel using ethyl acetate/hexane (4:1, v/v) to yield the product **5** as a slightly yellow solid (362 mg, 100%): ^{1}H NMR ($CDCl_3$, 500 MHz) δ 7.26 (s, 2H), δ 4.18 (t, J = 6.6 Hz, 2H), δ 3.24 (s, 2H), δ 2.26 (s, 3H), δ 1.87-1.76 (m, 2H), δ 1.55-1.46 (m, 2H), δ 1.40-1.19 (m, 26H), δ 0.90 (t, J = 6.9 Hz, 3H); ^{13}C ($CDCl_3$, 75 MHz): δ 160.42, 135.16, 132.74, 116.39, 81.24, 79.70, 74.52, 31.93, 30.25, 29.71, 29.69, 29.64, 29.47, 29.37, 25.92, 22.70, 20.27, 14.12; Cal'd for $C_{27}H_{40}O$ $[M+H]^+$ 381.3152; Found 381.3153.

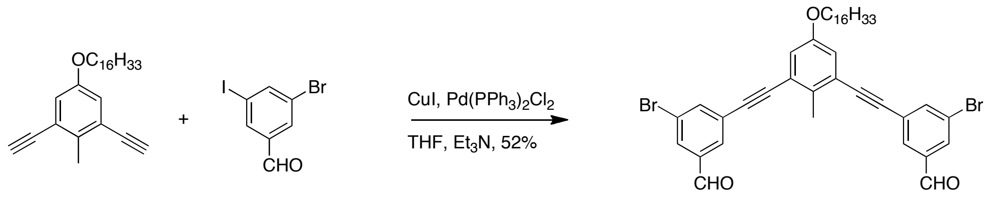

5 **2b**

Compound 2b: The general procedure for Sonogashira cross coupling was followed.[2] Using compound **5** (152 mg, 0.40 mmol), 3-bromo-5-iodo-benzaldyhyde (286 mg, 0.92 mmol), $Pd(PPh_3)_2Cl_2$ (17 mg, 0.02 mmol), CuI (1.5 mg, 0.008 mmol), triethylamine (4 mL), and THF (1 mL), compound **2b** was obtained as a colorless solid (155 mg, 52%): ^{1}H NMR ($CDCl_3$, 500 MHz) δ 9.98 (s, 2H), δ 8.00 (t, J = 1.7 Hz, 2H), δ 7.95 (t, J = 1.5 Hz, 2H), δ 7.91 (t, J = 1.7 Hz, 2H), δ 7.35 (s, 2H), δ 4.28 (t, J = 6.3 Hz, 2H), δ 2.34 (s, 3H), δ 1.93-1.84 (m, 2H), δ 1.63-1.58 (m, 2H), δ 1.40-1.14 (m, 26H), 0.89 (t, J = 6.9 Hz, 3H); ^{13}C ($CDCl_3$, 75 MHz): δ 189.87, 159.61, 139.34, 137.82, 134.92, 133.20, 131.70, 131.35, 126.33, 123.21, 116.57, 90.51, 88.51, 75.00, 31.93, 30.61, 29.73, 29.71, 29.69, 29.67, 29.37, 26.49, 22.70, 20.40, 14.14; Cal'd for $C_{41}H_{46}Br_2O_3$ $[M+Li]^+$ 751.1974; Found 751.1967.

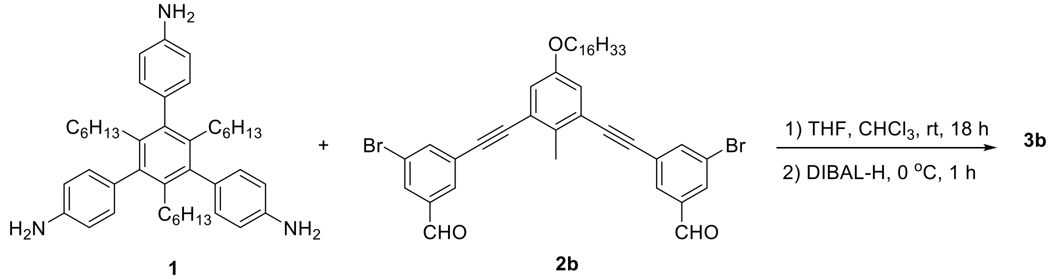

Cage 3b: To a schlenk tube was added compound **2b** (37 mg, 0.05 mmol) and compound **1** (20 mg, 0.033 mmol) in $CHCl_3$ (11 mL) under nitrogen atmosphere. TFA (0.38 µL, 0.005 mmol) in $CHCl_3$ (100 µL) was then added dropwise and the reaction was stirred at rt for 18h. The reaction mixture was cooled at 0 °C and DIBAL (1.24 mL, 1.0 M solution in CH_2Cl_2, 1.24 mmol) was added. The clear solution was stirred at 0 °C for 1h. Saturated

$NaHCO_3$ was added and the mixture was stirred at rt for 30 min. The mixture was extracted with $CHCl_3$ (3 x 50 mL) and the combined organic extracts were dried over anhydrous Na_2SO_4 and concentrated. The crude product was purified by flash column chromatography using CH_2Cl_2 as the eluent to afford the product **3b** as a white solid (13 mg, 24%): ^{1}H NMR (CD_2Cl_2, 500 MHz) δ 7.54 (s, 12H), δ 7.47 (s, 6H), δ 7.27 (s, 6H), δ 7.06-6.87 (m, 12H), δ 6.68-6.48 (m, 12H), δ 4.37 (s, 12H), δ 4.30 (s, 6H), δ 4.19 (t, J = 6.2 Hz, 6H), δ 2.28 (s, 6H), δ 2.02-1.94 (m, 12H), 1.86-1.78 (m, 6H), δ 1.60-0.63 (m, 153H); ^{13}C (CD_2Cl_2, 101 MHz): δ 159.47, 145.69, 142.82, 139.71, 138.94, 134.17, 133.10, 132.40, 130.88, 129.97, 128.73, 125.17, 122.39, 116.89, 91.65, 86.74, 74.64, 47.33, 31.90, 31.58, 30.73, 30.54, 30.47, 30.02, 29.70, 29.67, 29.64, 29.35, 29.29, 26.42, 22.68, 22.06, 20.09, 13.90, 13.86; MS (MALDI) Cal'd for $C_{207}H_{252}Br_6N_6O_3$ $[M+2Li]^+$ 3357.53; Found 3357.19.

Palladium nanoparticle growth

All reactions were carried out on a 4-8 mg scale with respect to cage **3a**. A solution of K_2PdCl_4 (1.8 mg, 0.0055 mmol) in deionized H_2O (0.5 mL) was stirred and tetraoctylammoniumbromide (TOAB) (31 mg, 0.056 mmol) in CH_2Cl_2 (0.5 mL) was added. The mixture was stirred until all Pd(II) was transferred into organic phase (~25 min). A solution of cage **3a** (4.0 mg, 0.0011 mmol) in CH_2Cl_2 (0.5 mL) was added and the mixture was stirred at rt for 30-45 min and then subsequently reduced with an aqueous solution of $NaBH_4$ (8.0 mg, 0.21 mmol) in deionized H_2O (0.5 mL). The resulting organic layer had the characteristic dark color of PdNP and contained no precipitate. After

separation, the aqueous layer was washed with CH_2Cl_2 (2 x 1 mL). The organic fractions were combined and concentrated to ca. 0.2 mL without the application of heat. The remaining organic solution (0.2 mL) was then transferred dropwise to a solution of ethanol (5-10 mL) to induce the precipitation of PdNP@**3a** complexes. The precipitates were separated by filtration. The resulting complexes are soluble in common organic solvents and stable in solution at room temperature.

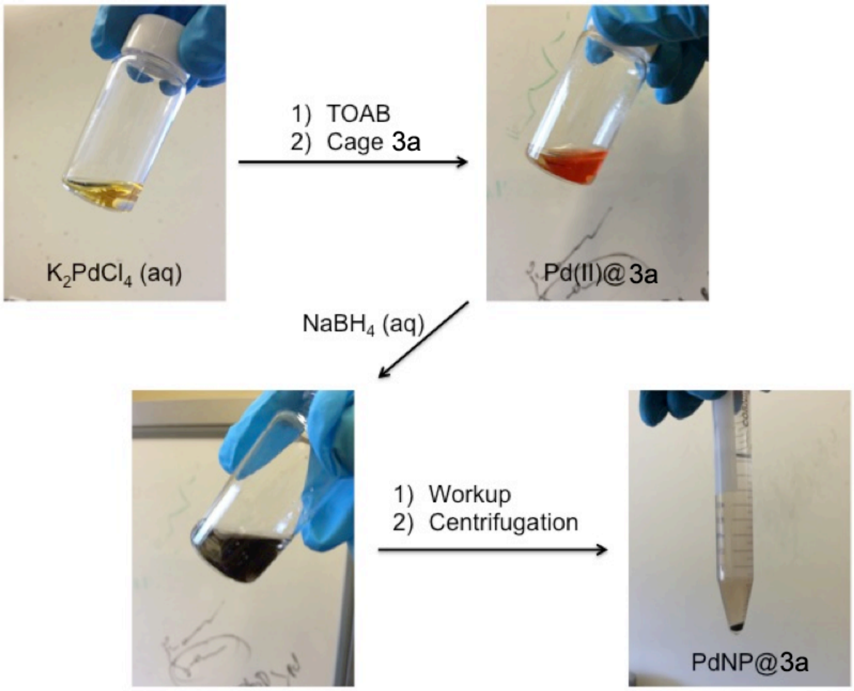

Figure S1. Synthesis of PdNP@**3a**

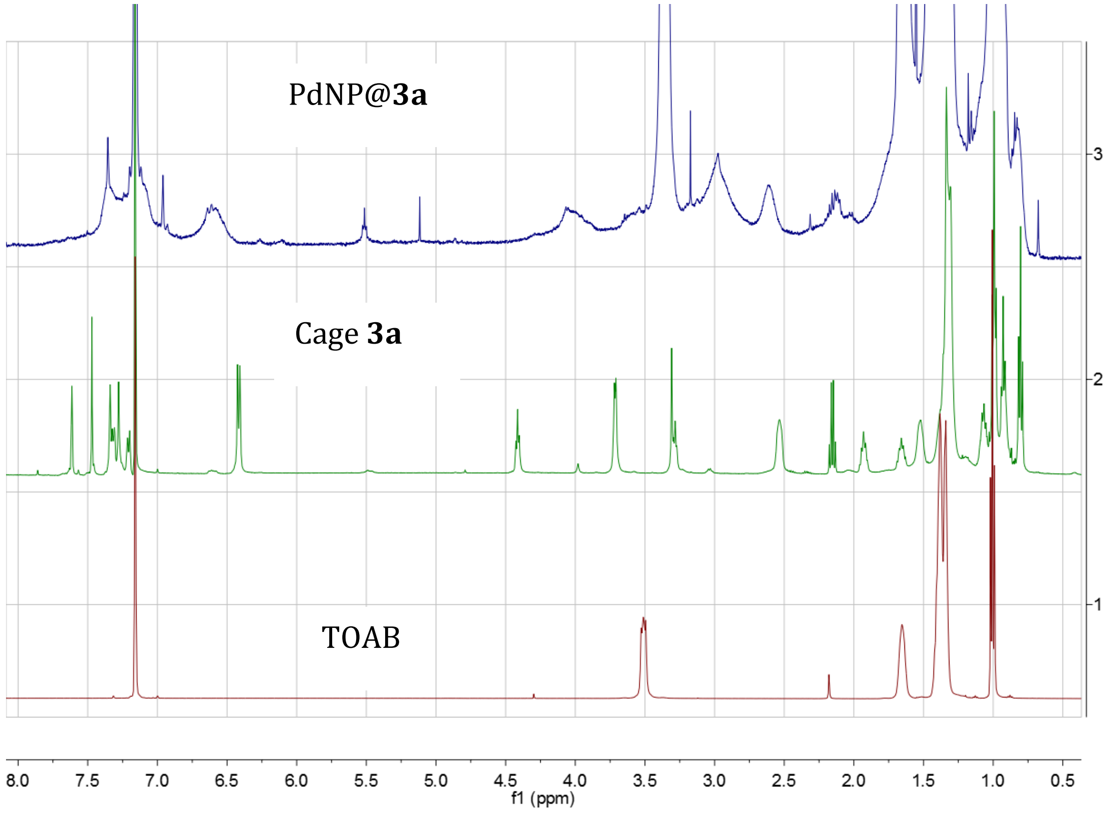

Figure S2. ^{1}H NMR spectra of TOAB, cage **3a**, and PdNP@**3a**.

General procedure for Suzuki coupling. To a flame-dried 10 mL microwave synthesizer reaction vessel was added: aryl halide (0.057 mmol), phenylboronic acid (0.087 mmol), and Na_2CO_3 (0.17 mmol). The vessel was sealed using a silicon cap with septum and further secured using a piece of tightly wrapped electrical tape. After degassing the vessel under vigorous vacuum/refill were added H_2O (200 μL) and PdNP@**3a** (we assume all the Pd were included in the cage) in toluene (0.4 mg in 2.0 mL) via syringe. The solution was degassed three times, transferred to the microwave reactor, and reacted using the following

conditions: 30 minutes, 100 °C (entries 1 and 2), or 140 °C (entries 3-6), 130 PSI, 50 W. After the reaction was complete, the solution was cooled to rt, poured into H_2O (4 mL) and extracted with $CHCl_3$ (3 x 2 mL). The organic fractions were combined, dried over anhydrous Na_2SO_4, and concentrated under reduced pressure.

3. GPC Graph of Cage 3a and 3b

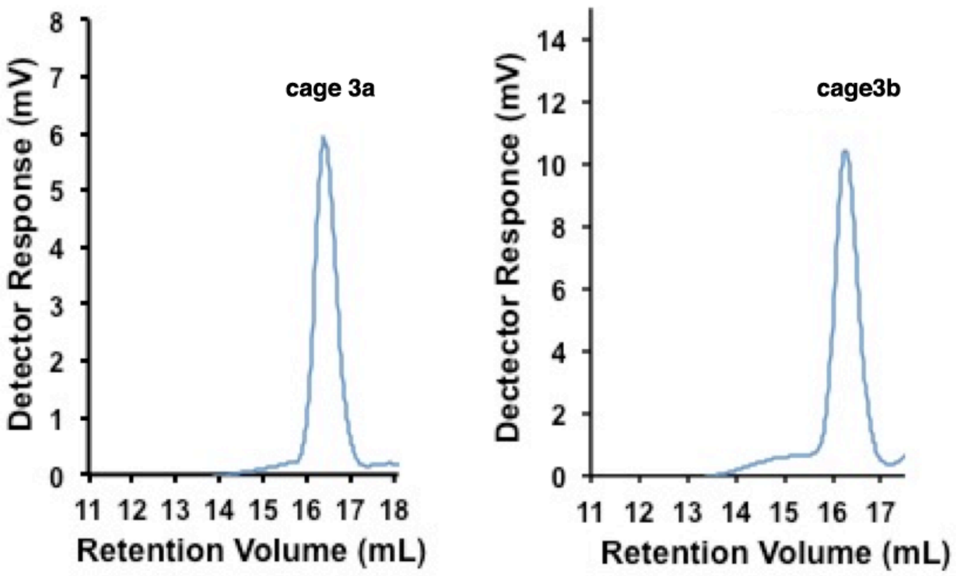

4. Transmission Electron Microscopy (TEM)

Imaging was performed using an FEI Tecnai F20 IVEM 200kV high resolution TEM. The solution of samples in CH_2Cl_2 was drop cast onto carbon-coated 300 mesh copper grids (CF300-Cu) from Ted Pella. Particle size data of over 500 particles were obtained by analyzing TEM images using software ImageJ.

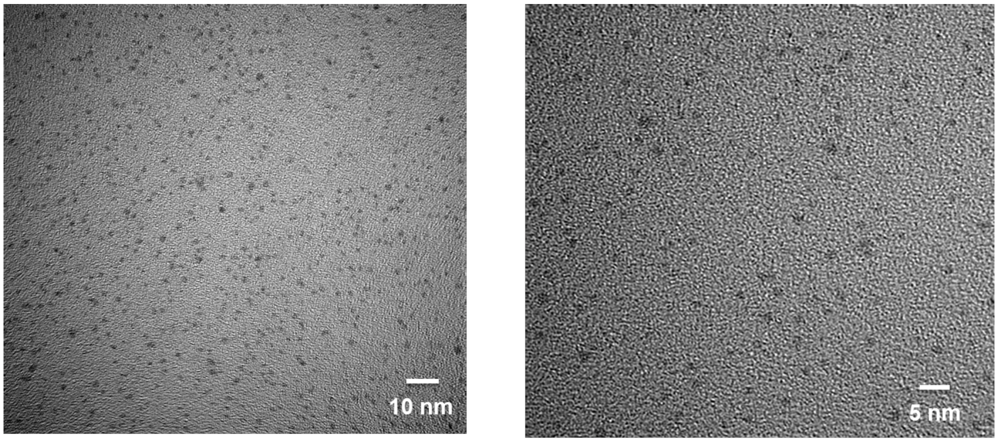

Figure S3. Representative TEM images.

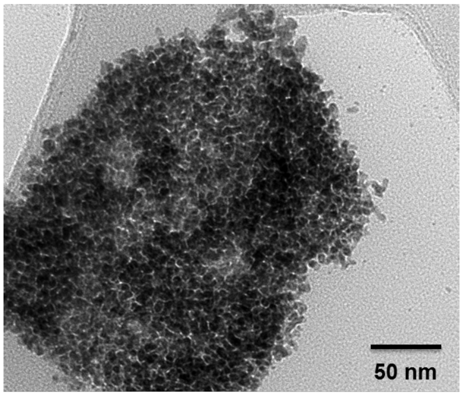

Figure S4. Control Experiment in the absence of cage **3a**.

5. Energy-dispersive X-ray spectroscopy (EDS)

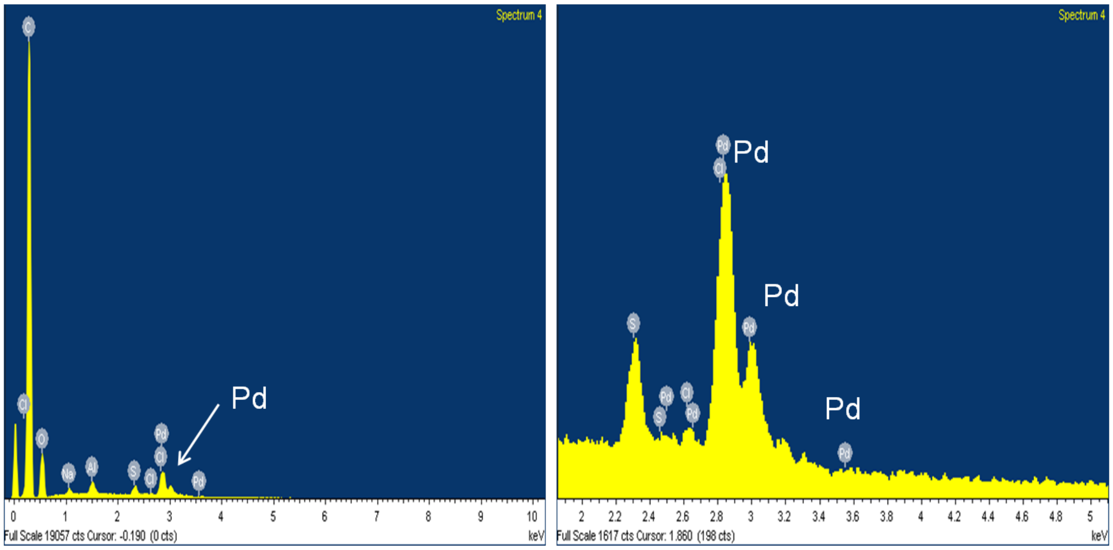

Figure S5. EDS spectra of PdNP@**3a** complex.

6. Diffusion NMR experiment

Diffusion ordered spectroscopy (DOSY) NMR was performed on a Varian Inova 500 MHz NMR spectrometer. Both samples of cage **3a** and PdNP@**3a** (with a small amount of TOAB to maintain good solubility in C_6D_6) were measured using C_6D_6 as the solvent at concentrations of 5 mM. The effective hydrodynamic radius R was calculated using the Strokes-Einstein equation: $D = (k_BT)/(6\pi\eta r)$, where D is the diffusion coefficient, k_B is the Boltzmann constant, T is absolute temperature (23 ^{o}C), and η is the viscosity of C_6D_6 (0.65 mPa·s). The measured diffusion coefficient for cage **3a** was 2.5±0.5x10^{-10} m^{2}s^{-1} and for PdNP@**3a** was 2.4±0.5x10^{-10} m^{2}s^{-1} thus giving hydrodynamic radius of 13Å±1.3 Å and 14 Å±1.3 Å, respectively.

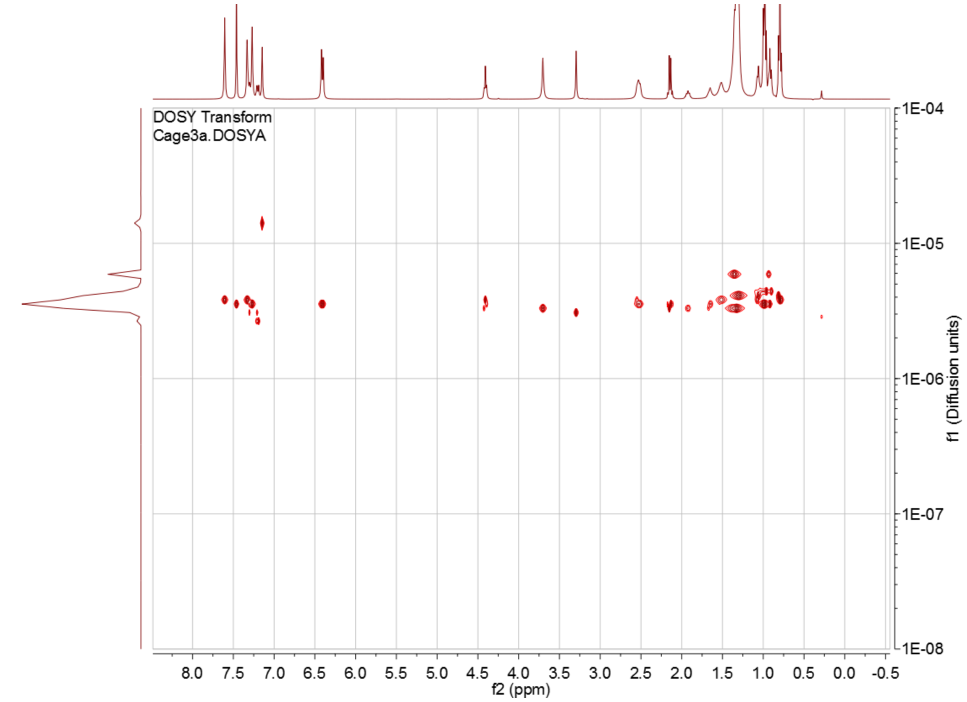

Figure S6. ^{1}H 2D-DOSY NMR spectrum of cage **3a** in C_6D_6. A Diffusion cross-peak for the solvent (7.16 ppm) is also present.

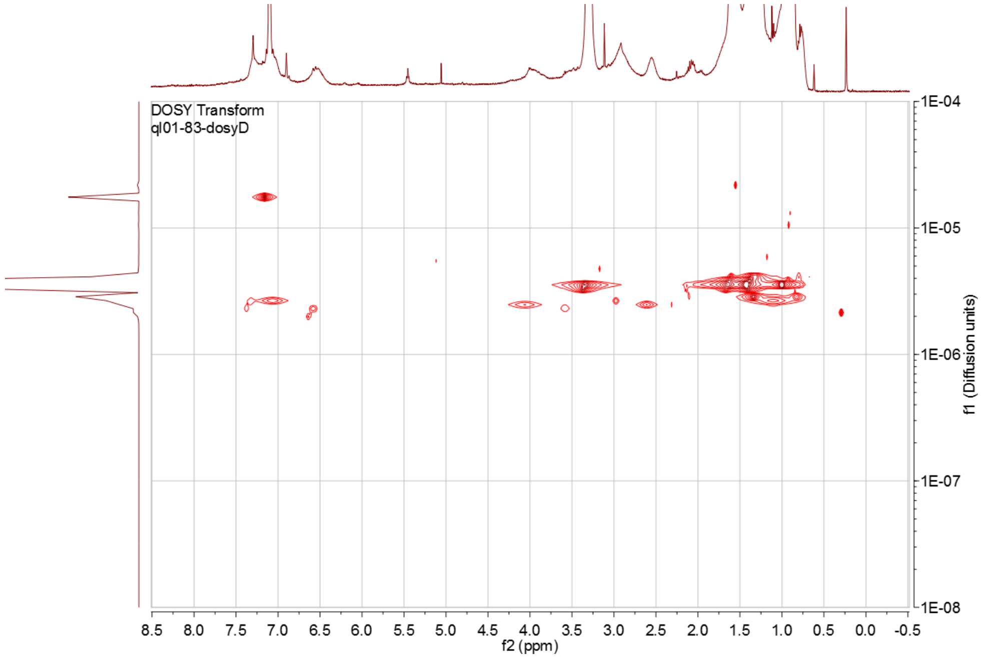

Figure S7. ^{1}H 2D-DOSY NMR spectrum of PdNP@**3a** in C_6D_6. Diffusion cross-peaks for the solvent (7.16 ppm) and TOAB are also present.

7. Recycle test of PdNP@3a.[a]

Table S1: Recycle test of PdNP@**3a** in Suzuki-Miyauru coupling.

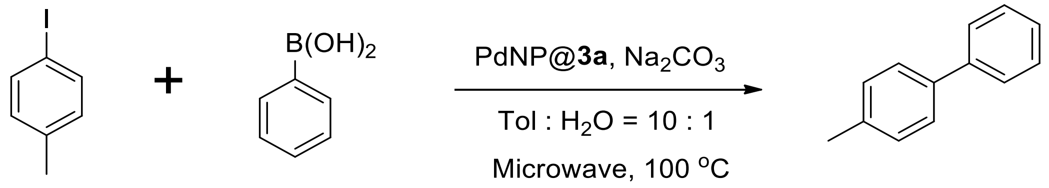

**Batch** [b]	**Time (min)**	**Yield [%]** [c]
1	15	99
2	15	99
3	15	98
4	15	96

[a] Reaction conditions: iodotoluene (0.057 mmol), phenylboronic acid (0.087 mmol), Na_2CO_3 (0.17 mmol), Pd catalyst (0.57 µmol, 1.0 mol%).[b] For batches 2-4, iodotoluene (0.057 mmol), phenylboronic acid (0.087 mmol) and Na_2CO_3 (0.057 mmol) was added into the same reaction vessel after the previous batch. [c] Yields are based on ^{1}H NMR analysis of the crude products.

7. NMR spectra of compounds

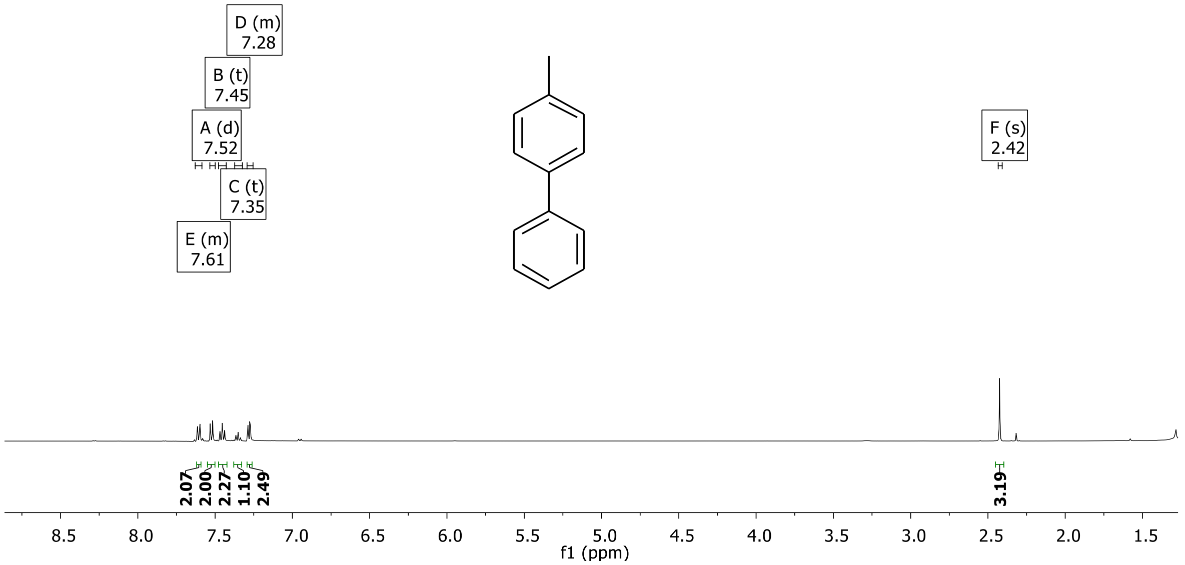

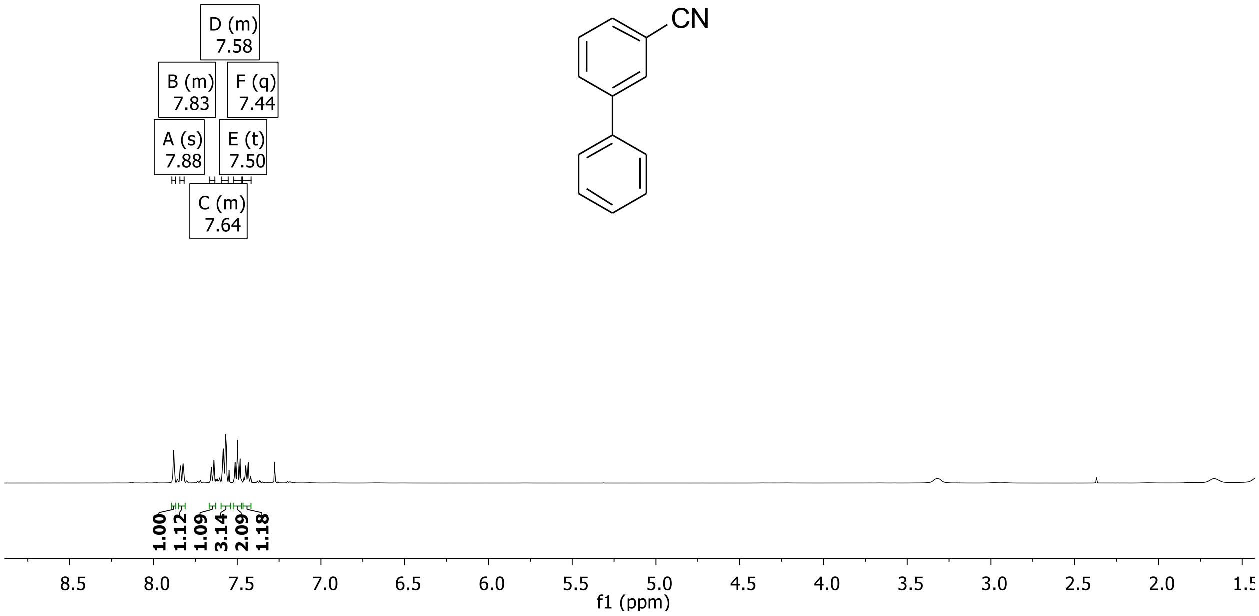

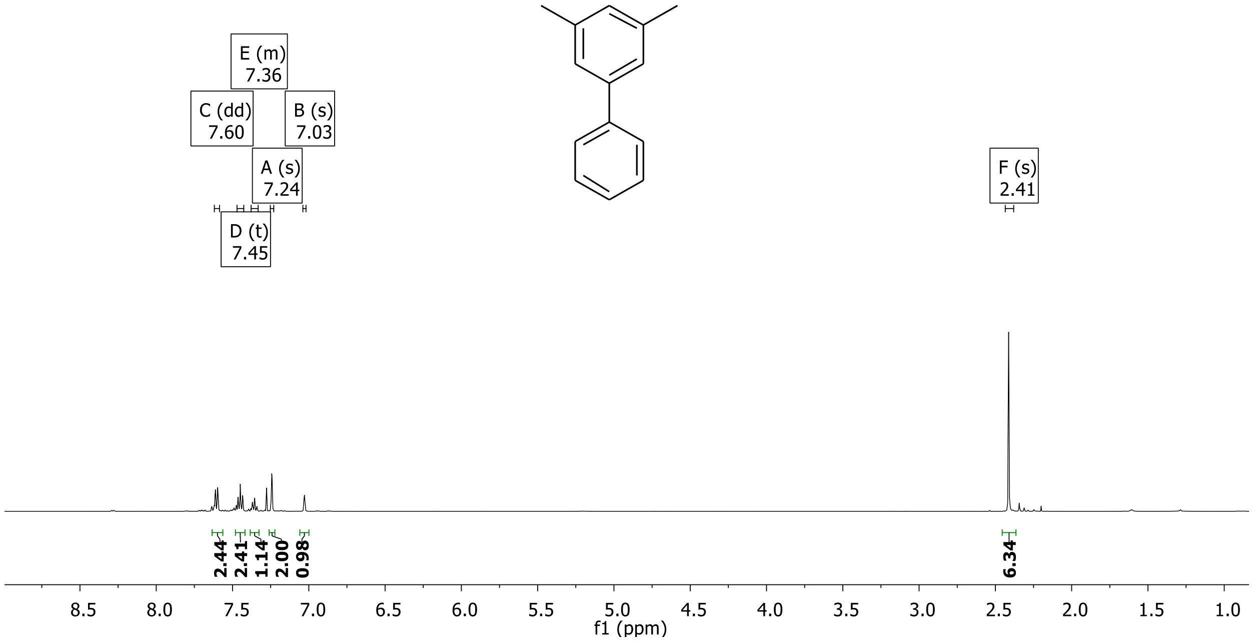
E (m)
7.36
C (dd)
7.60
B (s)
7.03
A (s)
7.24
D (t)
7.45
F (s)
2.41
2.44
2.41
1.14
2.00
0.98
6.34
8.5 8.0 7.5 7.0 6.5 6.0 5.5 5.0 4.5 4.0 3.5 3.0 2.5 2.0 1.5 1.0
f1 (ppm)

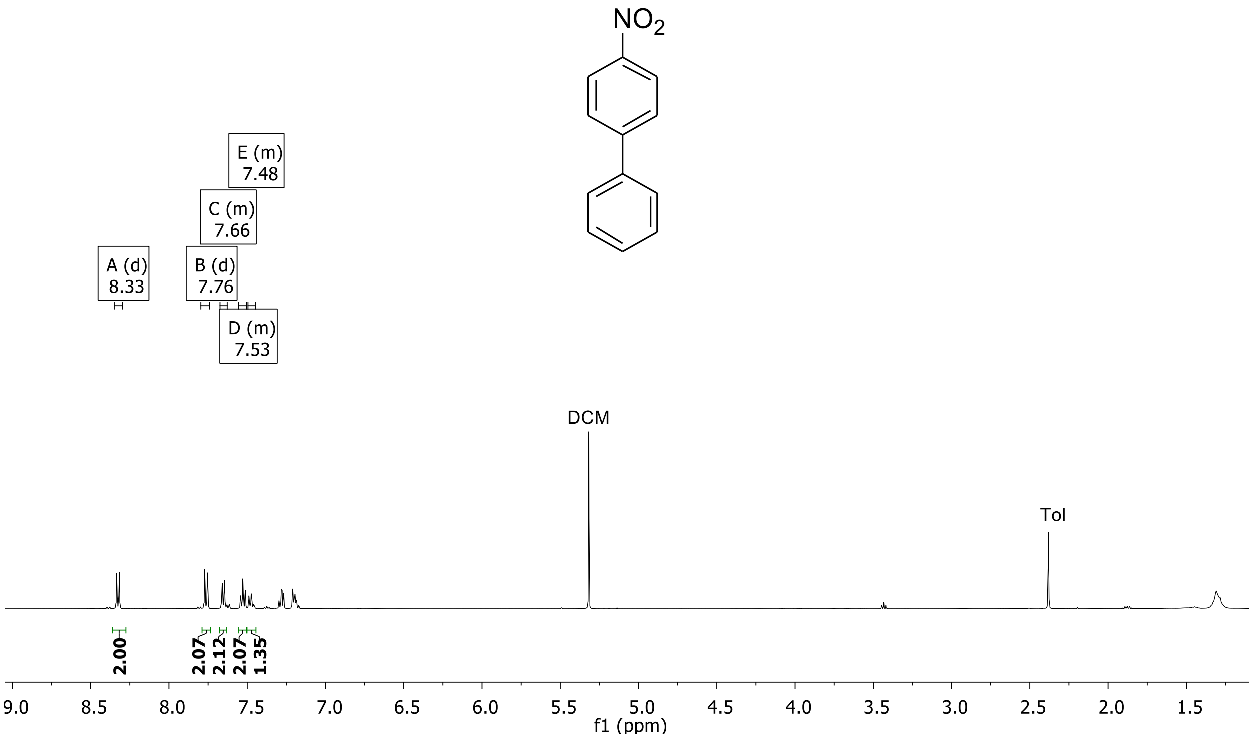
NO2
E (m)
7.48
C (m)
7.66
A (d)
8.33
B (d)
7.76
D (m)
7.53
DCM
Tol
2.00
2.07
2.12
2.07
1.35
9.0 8.5 8.0 7.5 7.0 6.5 6.0 5.5 5.0 4.5 4.0 3.5 3.0 2.5 2.0 1.5
f1 (ppm)

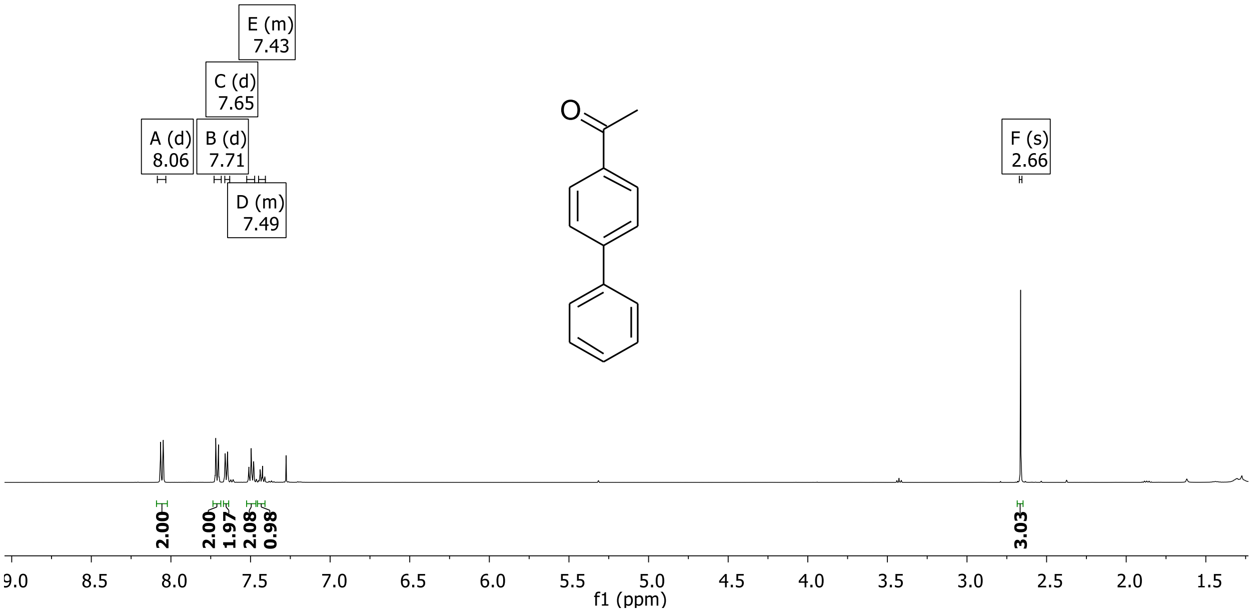
E (m)
7.43
C (d)
7.65
A (d)
8.06
B (d)
7.71
D (m)
7.49
O
F (s)
2.66
2.00
2.00
1.97
2.08
0.98
3.03
9.0
8.5
8.0
7.5
7.0
6.5
6.0
5.5
5.0
4.5
4.0
3.5
3.0
2.5
2.0
1.5
f1 (ppm)

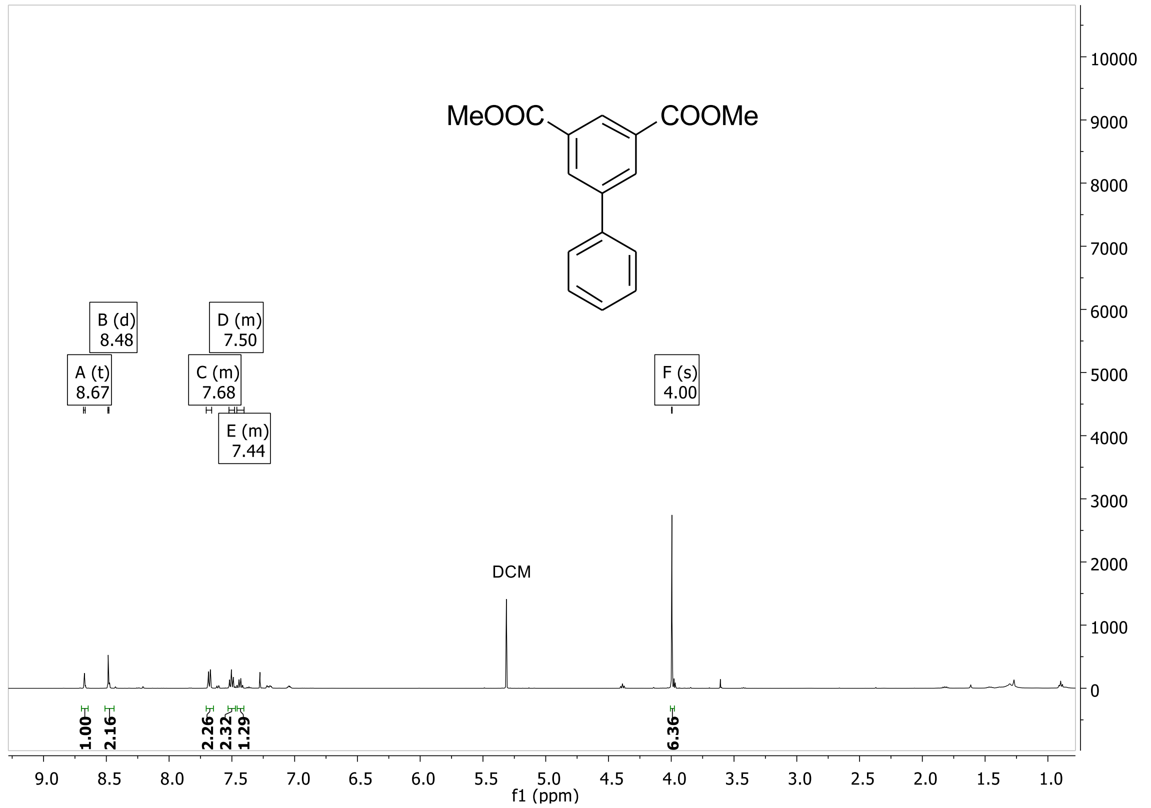
MeOOC
COOMe
B (d)
8.48
D (m)
7.50
A (t)
8.67
C (m)
7.68
F (s)
4.00
E (m)
7.44
DCM
1.00
2.16
2.26
2.32
1.29
6.36
10000
9000
8000
7000
6000
5000
4000
3000
2000
1000
0
9.0
8.5
8.0
7.5
7.0
6.5
6.0
5.5
5.0
4.5
4.0
3.5
3.0
2.5
2.0
1.5
1.0
f1 (ppm)

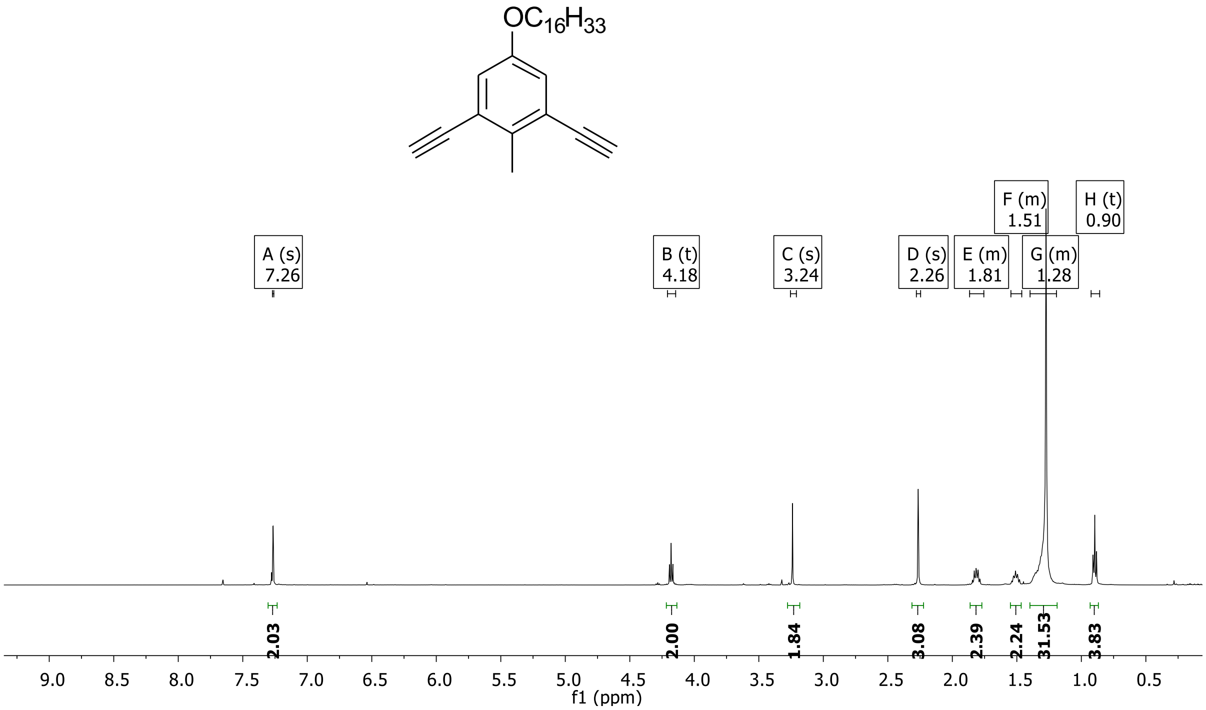
OC16H33
A (s)
7.26
B (t)
4.18
C (s)
3.24
D (s)
2.26
E (m)
1.81
F (m)
1.51
G (m)
1.28
H (t)
0.90
2.03
2.00
1.84
3.08
2.39
2.24
31.53
3.83
9.0 8.5 8.0 7.5 7.0 6.5 6.0 5.5 5.0 4.5 4.0 3.5 3.0 2.5 2.0 1.5 1.0 0.5
f1 (ppm)

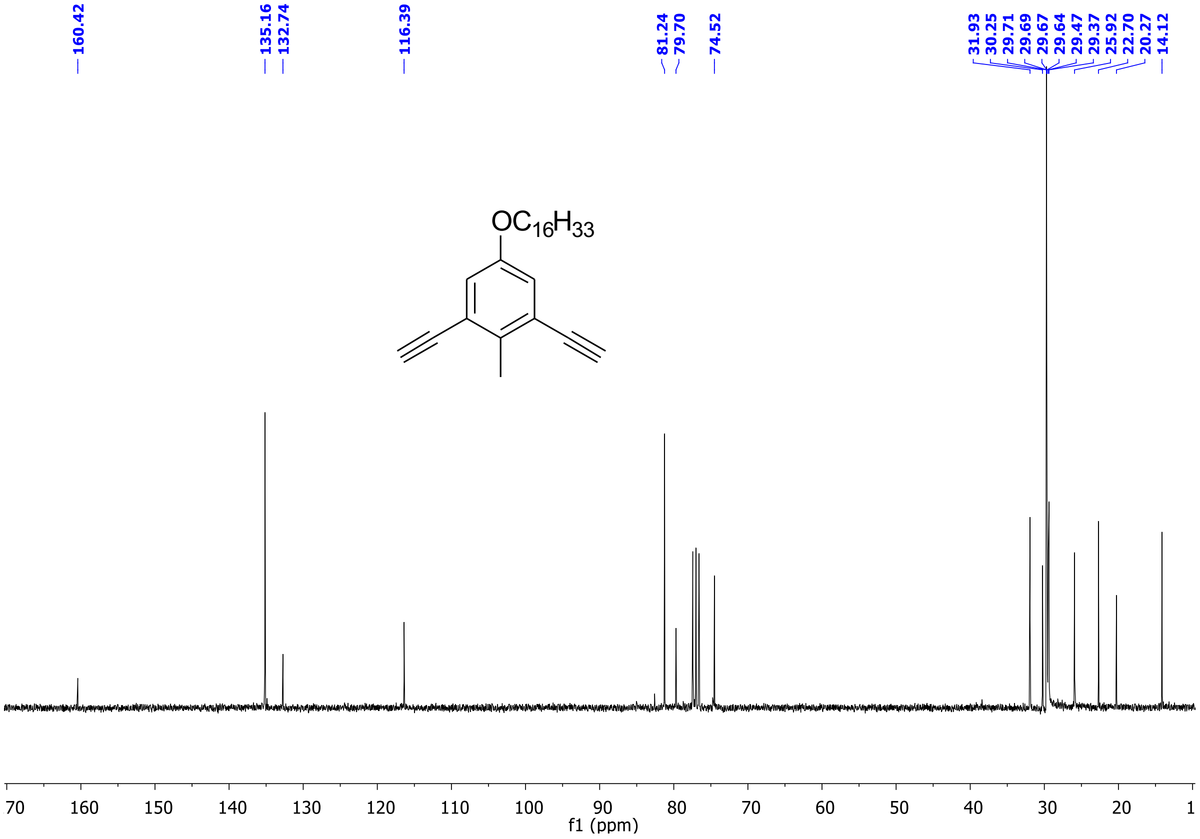
160.42
135.16
132.74
116.39
81.24
79.70
74.52
31.93
30.25
29.71
29.69
29.67
29.64
29.47
29.37
25.92
22.70
20.27
14.12
OC16H33
70 160 150 140 130 120 110 100 90 80 70 60 50 40 30 20 1
f1 (ppm)

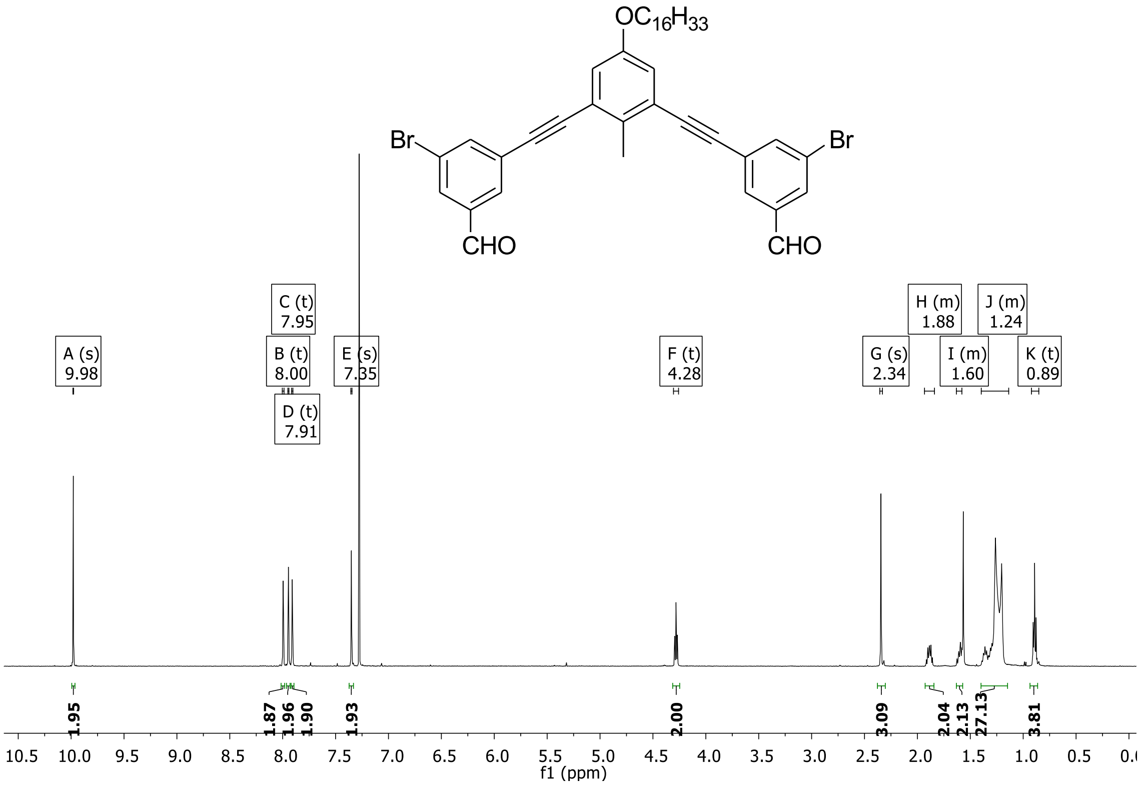
OC16H33
Br
Br
CHO
CHO
A (s) 9.98
B (t) 8.00
C (t) 7.95
D (t) 7.91
E (s) 7.35
F (t) 4.28
G (s) 2.34
H (m) 1.88
I (m) 1.60
J (m) 1.24
K (t) 0.89
1.95
1.87
1.96
1.90
1.93
2.00
3.09
2.04
2.13
27.13
3.81
f1 (ppm)

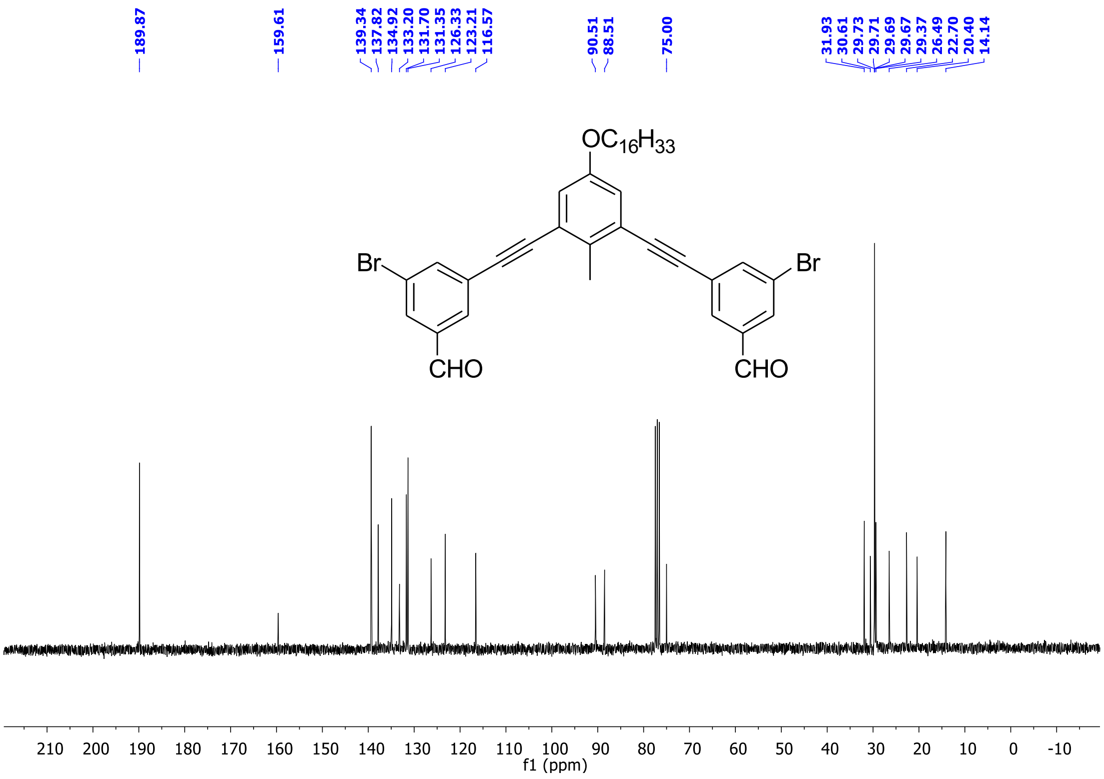
189.87
159.61
139.34
137.82
134.92
133.20
131.70
131.35
126.33
123.21
116.57
90.51
88.51
75.00
31.93
30.61
29.73
29.71
29.69
29.67
29.37
26.49
22.70
20.40
14.14
OC16H33
Br
Br
CHO
CHO
f1 (ppm)

Cage 3b

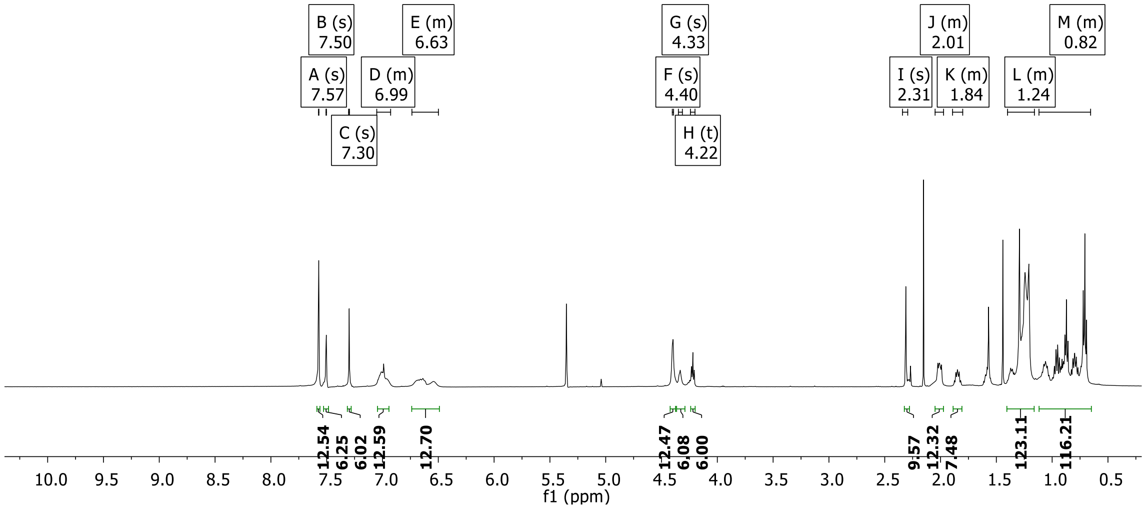

B (s) 7.50
E (m) 6.63
G (s) 4.33
J (m) 2.01
M (m) 0.82
A (s) 7.57
D (m) 6.99
F (s) 4.40
I (s) 2.31
K (m) 1.84
L (m) 1.24
C (s) 7.30
H (t) 4.22
12.54
6.25
6.02
12.59
12.70
12.47
6.08
6.00
9.57
12.32
7.48
123.11
116.21
f1 (ppm)

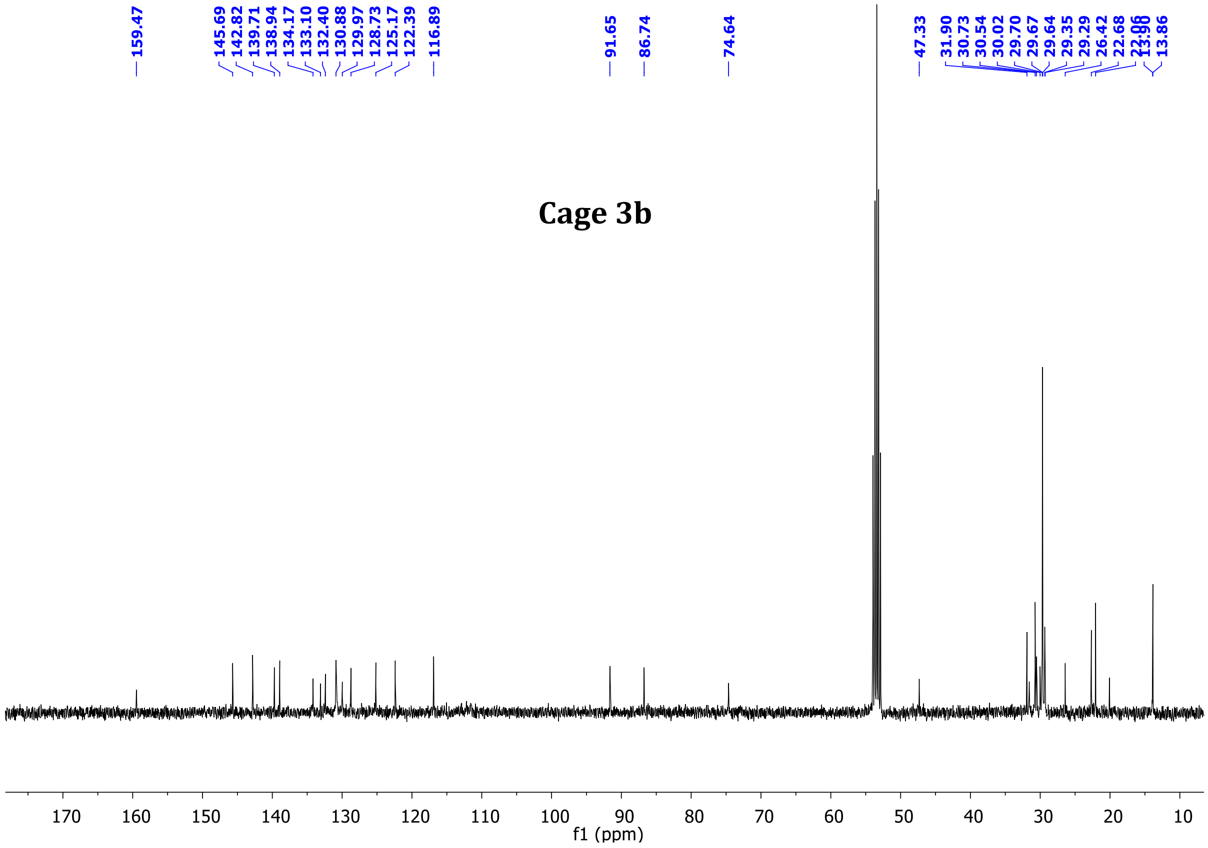

159.47
145.69
142.82
139.71
138.94
134.17
133.10
132.40
130.88
129.97
128.73
125.17
122.39
116.89
91.65
86.74
74.64
47.33
31.90
30.73
30.54
30.02
29.70
29.67
29.64
29.35
29.29
26.42
22.68
13.86
Cage 3b
f1 (ppm)

8. References

1. R. McCaffrey, H. Long, Y. Jin, A. Sanders, W. Park, W. Zhang, *J. Am. Chem. Soc.* **2014**, 136, 1782.

2. (a) K. Sonogashira, Y. Tohda, N. Hagihara, *Tetrahedron Lett*. **1975**, 4467-4470; (b) K. Sonogashira, T. Yatake, Y. Tohda, S. Takahashi, N. Hagihara, *J. Chem. Soc. Chem. Commun.* **1977**, 291-292.
